# Explore the genetics of weedy traits using rice 3K database

**DOI:** 10.1186/s40529-020-00309-y

**Published:** 2021-01-12

**Authors:** Yu-Lan Lin, Dong-Hong Wu, Cheng-Chieh Wu, Yung-Fen Huang

**Affiliations:** 1 Department of Agronomy, National Taiwan University, No. 1, Sec. 4, Roosevelt Rd, Da’an Dist., Taipei, 10617 Taiwan; 2 Taiwan Agricultural Research Institute, Council of Agriculture, Executive Yuan, No. 189, Zhongzheng Rd, Wufeng Dist, Taichung City, 41362 Taiwan; 3 Institute of Plant and Microbial Biology, Academia Sinica, Taipei, 11529 Taiwan; 4 Institute of Plant Science, National Taiwan University, No. 1, Sec. 4, Roosevelt Rd, Da’an Dist., Taipei, 10617 Taiwan

**Keywords:** *Oryza sativa* L., Weedy rice (WR), 3K Rice Genome Project (3K-RGP), Genome-wide association study (GWAS), SNP-Seek

## Abstract

**Background:**

Weedy rice, a conspecific weedy counterpart of the cultivated rice (*Oryza sativa* L.), has been problematic in rice-production area worldwide. Although we started to know about the origin of some weedy traits for some rice-growing regions, an overall assessment of weedy trait-related loci was not yet available. On the other hand, the advances in sequencing technologies, together with community efforts, have made publicly available a large amount of genomic data. Given the availability of public data and the need of “weedy” allele mining for a better management of weedy rice, the objective of the present study was to explore the genetic architecture of weedy traits based on publicly available data, mainly from the 3000 Rice Genome Project (3K-RGP).

**Results:**

Based on the results of population structure analysis, we have selected 1378 individuals from four sub-populations (*aus*, *indica*, *temperate japonica*, *tropical japonica*) without admixed genomic composition for genome-wide association analysis (GWAS). Five traits were investigated: awn color, seed shattering, seed threshability, seed coat color, and seedling height. GWAS was conducted for each sub-population × trait combination and we have identified 66 population-specific trait-associated SNPs. Eleven significant SNPs fell into an annotated gene and four other SNPs were close to a putative candidate gene (± 25 kb). SNPs located in or close to *Rc* were particularly predictive of the occurrence of seed coat color and our results showed that different sub-populations required different SNPs for a better seed coat color prediction. We compared the data of 3K-RGP to a publicly available weedy rice dataset. The profile of allele frequency, phenotype-genotype segregation of target SNP, as well as GWAS results for the presence and absence of awns diverged between the two sets of data.

**Conclusions:**

The genotype of trait-associated SNPs identified in this study, especially those located in or close to *Rc*, can be developed to diagnostic SNPs to trace the origin of weedy trait occurred in the field. The difference of results from the two publicly available datasets used in this study emphasized the importance of laboratory experiments to confirm the allele mining results based on publicly available data.

## Background

Weedy rice (WR), a conspecific weedy counterpart of the cultivated rice (*Oryza sativa* L.), is probably the most intractable problem to deal with in rice-growing countries. Herbicide inapplicability, as well as the morphological resemblance, have made WR management an uphill struggle for rice farmers around the world (Olofsdotter et al. 2000). Each year, WR causes important economic loss in rice production due to the reduction in yield and in grain quality (Ziska et al. 2015). Although sharing a high level of genetic similarities with cultivated rice, WR is often characterized by some wild-like traits, such as rapid seedling growth, the presence of awns, high degree of shattering, red seed coat color, and strong dormancy, which together contribute to increasing WR’s ability to survive in the field (Ziska et al. 2015). Indeed, such “weedy” traits and the domestication syndromes are like the two sides of a coin. While cultivated rice has undergone intense selection against shattering to reduce grain loss during harvest, shattering habit of WR facilitates seed dispersal in the field (Delouche et al. 2007a). WR possess awns to avoid grain predation by animals (Delouche et al. 2007b) while cultivated rice is mainly characterized by awn-less grains. Red seed coat, or pericarp, is common to WR after which “weedy red rice” is named although colored seed coat is also observed in some cultivars. A number of identified genes know to contribute to the pigmentation of rice pericarp, among which a major factor is *Rc*, a bHLH protein involving in proanthocyanidin synthesis (Furukawa et al. 2007; Sweeney et al. 2006). Potentially descended from cultivated ancestors, the weedy traits that WR possesses are likely to be derived from standing variation in wild or cultivated rice (Huang et al. 2018; Vigueira et al. 2019).

The occurrence of WR is generally acknowledged as polyphylogenetic. For example, the U.S. has suffered serious WR infestation which mainly comprised of two morphologically different ecotypes, the black-hull awned (BHA) and the straw awn-less (SH) populations (Londo and Schaal 2007). Studies based on phylogenetic analysis through nucleic and cytoplasmic DNA evidence suggested that BHA and SH were possibly derived from *aus* and *indica*, respectively (Reagon et al. 2010). None of the cultivars grown in the U.S. belongs to these two groups, indicating that BHA and SH may be originated from stock seeds contamination or from escaped breeding materials (Olsen et al. 2007). On the other hand, WR in northern China is most likely originated from hybridization between local *japonica* landraces whereas WR in southern China was genetically similar to *indica,* the most cultivated sub-species in the south of China (Sun et al. 2019). Previous studies have also implied that wild rice may have participated in the formation of WR through inter-specific hybridization (Song et al. 2014; Vigueira et al. 2019). To date, most of the WR genetic studies focused on its occurrence using whole genome sequencing (Huang et al. 2018; Li et al. 2017; Qiu et al. 2017; Vigueira et al. 2019). Loci related to weedy traits were identified through genome-wide selection signature analysis with a focus on known domestication-related genes such as *Bh4*, *PROG1*, *Rc*, *sh4* (Huang et al. 2018; Li et al. 2017; Qiu et al. 2017). The actual phenotype-genotype studies on weedy traits were relatively few (Nguyen et al. 2019; Ye et al. 2013). However, the knowledge on weedy trait genetics would help in a better WR management.

Rapid development of next-generation sequencing (NGS) technologies has brought plant genetics into a new era (Bräutigam and Gowik 2010; Varshney et al. 2014). Large amounts of data have been generated and shared openly with the scientific community. In rice, in addition to the reference genome (cv. Nipponbare, IRGSP-1.0), many genomic tools are available. SNP-Seek database (https://snp-seek.irri.org/) is a major outcome of the 3000 rice genome project (3K-RGP). SNP-Seek harbors genotypic and phenotypic data for more than 3000 accessions of cultivated rice (Mansueto et al. 2016a, b), has provided valuable resources for genomic discovery of *Oryza sativa*, and has opened the doors for large-scale association studies and for an efficient molecular breeding (Angira et al. 2019; Kumar et al. 2020; Leung et al. 2015; Mansueto et al. 2016b; Tang et al. 2019; Tatarinova et al. 2016). Indeed, whole genome association results based on the full 3K-RGP data are available on SNP-Seek. Meanwhile, if one is interested in sub-population specific trait-marker association, a re-analysis may be necessary.

Given the availability of public data and the need of allele mining for weedy traits in view of a better WR management, the objective of the present study was to explore the genetic architecture of rice weedy traits based on publicly available data. We first investigated the abundant data from 3K-RGP and then cross-checked the results with another WR panel, also obtained from public resources.

## Methods

### Source data

**Fig. 1:**
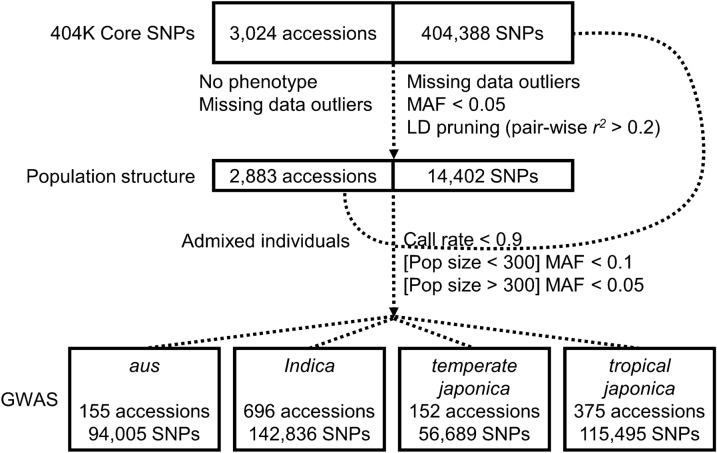
Workflow of 3K-RGP genotypic data preparation. The short sentences next to the dash lines indicate criteria to remove accessions or SNPs from previous data set. After population structure analysis, while the individuals were thinned, we went back to the 404K core SNPs to filter SNPs for GWAS

Both phenotypic data and genotypic data of the 3000 Rice Genome Project (3K-RGP) were downloaded from the Rice SNP-Seek Database (http://snp-seek.irri.org). The full set of phenotypes consisted of 47 agronomic traits recorded in ordinal scale between 1994 and 2010, maintained in the International Rice Genebank Collection Information System (IRGCIS). For our purpose, we have selected five traits which are often viewed as notable features distinguishing WR from cultivated rice (Meyer and Purugganan 2013): awn color (AWCO_REV), degree of panicle shattering (PSH), panicle threshability (PTH), seed coat color (SCCO_REV), and seedling height (SDHT_CODE). We chose to follow the trait abbreviation according to IRGCIS for an easier cross comparison between studies. For genotypic data, we have chosen the 404K core SNP set. The 404K core SNPs consisted of 404,388 bi-allelic SNPs across 3024 rice accessions, generated through a two-step linkage disequilibrium (LD) pruning procedure to reduce the number of markers at a minor allele frequency (MAF) > 0.01 and a call rate ≥ 0.8. Detailed data filtering process is described at http://snp-seek.irri.org/_download.zul. As different datasets were generated for different analyses, details for different datasets will be provided accordingly. Meanwhile, a summary genotypic data workflow can be found in Fig. 1.

To compare the trait-associated markers identified in rice 3K dataset to those identified in WR background, we have obtained phenotypes and sequence reads of 205 WR accessions from two studies (Li et al. 2017; Qiu et al. 2017), among which 198 were unique. These accessions mainly encompassed four different geographical regions within China (162 accessions), the U.S. (42 accessions), and South Korea (1 accession). Raw sequence reads were retrieved from NCBI (SRR4334499 and PRJNA295802). We performed variant calling in accordance with the SNP discovery pipeline described in Mansueto et al. (2016a, b): raw sequence reads were first mapped to *japonica* reference genome (cv. Nipponbare, IRGSP-1.0) via Burrows-Wheeler Alignment tool (BWA) version 0.7.17-r1188 (Li and Durbin 2009) with default settings after removing adapter sequences. The resulting alignments were processed using MarkDuplicates of Picard (http://broadinstitute.github.io/picard/) and IndelRealigner implemented in The Genome Analysis Toolkit (GATK) version 3.8-0-ge9d806836 (McKenna et al. 2010). Final variant calling from the 198 WR samples were performed using BCFtools version 1.10.2 (http://www.sanger.ac.uk/science/tools/samtools-bcftools-htslib). We used BEAGLE (Browning et al. 2018) to impute missing genotype with default parameters.

### Population structure and linkage disequilibrium

Population structure was inferred using both principle component analysis (PCA) and the model-based maximum likelihood approach implemented in software ADMIXTURE v1.23 (Alexander et al. 2009). Data for population structure inference was prepared as follows.

We first excluded accessions with no phenotypic data available for all five target traits. We then used PLINK v1.9 (Purcell et al. 2007) for the subsequent SNP filtering and pruning. We applied the 1.5× interquartile range to remove outliers in terms of missing rate at per accession level (> 0.08817) and at per SNP level (> 0.1408). SNPs with MAF ≥ 0.05 were retained. In order to generate independent SNP for population structure analysis, we pruned SNPs at a window of 50 SNPs. SNPs showing pair-wise *r*^*2*^ higher than 0.2 were removed and the pruning was advanced along the genome at a step of five SNPs. This resulted in a dataset of 2883 individuals × 14,462 SNPs (Fig. 1).

PCA was performed in GCTA (Yang et al. 2011). For ADMIXTURE, K = 1 to 15 were tested. Since the cross-validation error barely differed between K values, we defined K = 5 based on the prior knowledge on sub-populations within the rice 3K samples (Wang et al. 2018). To avoid the interference of population structure in marker-trait association, only accessions belonging clearly to one sub-population (defined as proportion of genome from a single source ≥ 0.8) were retained for further analyses. This resulted in a final of 1378 accessions divided into four sub-populations (Fig. 1): *aus* (155), *indica* (696), *temperate japonica* (152), and *tropical japonica* (375).

LD was estimated as the squared correlation coefficient of allele state (*r*^*2*^) within each sub-population using PLINK. Pairwise *r*^*2*^ values were computed between all SNPs within 300 kb along the same chromosome according to the study of Wang et al. (2018). Average *r*^*2*^ was calculated for each 1 kb-bin across the whole genome for graphical visualization.

### Genome-wide Association Study (GWAS)

Genotypic data for each sub-population were prepared from 404K core SNPs using different marker filtering criteria: for sub-populations contained fewer than 300 accessions, i.e., *aus* and *temperate japonica*, SNPs were excluded from GWAS if call rate < 0.9 and MAF < 0.1; for sub-population contained more than 300 accessions, i.e., *indica* and *tropical japonica*, SNPs were excluded from GWAS if call rate < 0.9 and MAF < 0.05. This gave a total number of 94,005 SNPs for *aus*, 142,836 SNPs for *indica*, 115,495 SNPs for *tropical japonica*, and 56,689 SNPs for *temperate japonica* (Fig. 1).

We used the Fixed and random model Circulating Probability Unification (FarmCPU) (Liu et al. 2016) for GWAS in our study. This method divides the multiple loci linear mixed model into two parts: a fixed effect model (FEM) and a random effect model (REM) which were used iteratively. FEM includes one-by-one marker testing; multiple associated markers, or pseudo quantitative trait nucleotide (pseudo QTN), were further treated as covariates to control false positives. To avoid the model over-fitting problem in FEM, the pseudo QTN were used to define the kinship in order to model individuals’ total genetic effect in REM. Through this two-stage iterative process, FarmCPU has not only increased statistical power but also reduced computation time while controlling both false positives and false negatives. The inclusion of covariate to control for population structure can increase the detection power and eliminate false positives than without the inclusion of covariates (Liu et al. 2016). Therefore, principle components (PC) generated from PCA were used as covariates. For each given trait within a sub-population, GWAS models including zero to five PC were compared with each other based on quantile–quantile (Q–Q) plots (expected p-values vs. observed p-values) to identify the best-fit model. Significant marker-trait association were identified using Bonferroni threshold at an error rate of 0.05.

### Cross-validation of associated SNPs

For each significant SNP, we retrieved all genes located within ± 100-kb flanking region and defined candidate genes using the gene annotation from the Rice Annotation Project Database (RAP-DB, http://rapdb.dna.affrc.go.jp/) (Sakai et al. 2013). For trait-associated SNPs, contingency tables between SNP alleles and phenotype were made and visually inspected to examine the association between genotype and phenotype. Based on the GWAS results of 3K RGP, we verified the associated variants using WR data through the inspection of genotype–phenotype distribution.

## Results

### Population structure and LD analysis

**Fig. 2:**
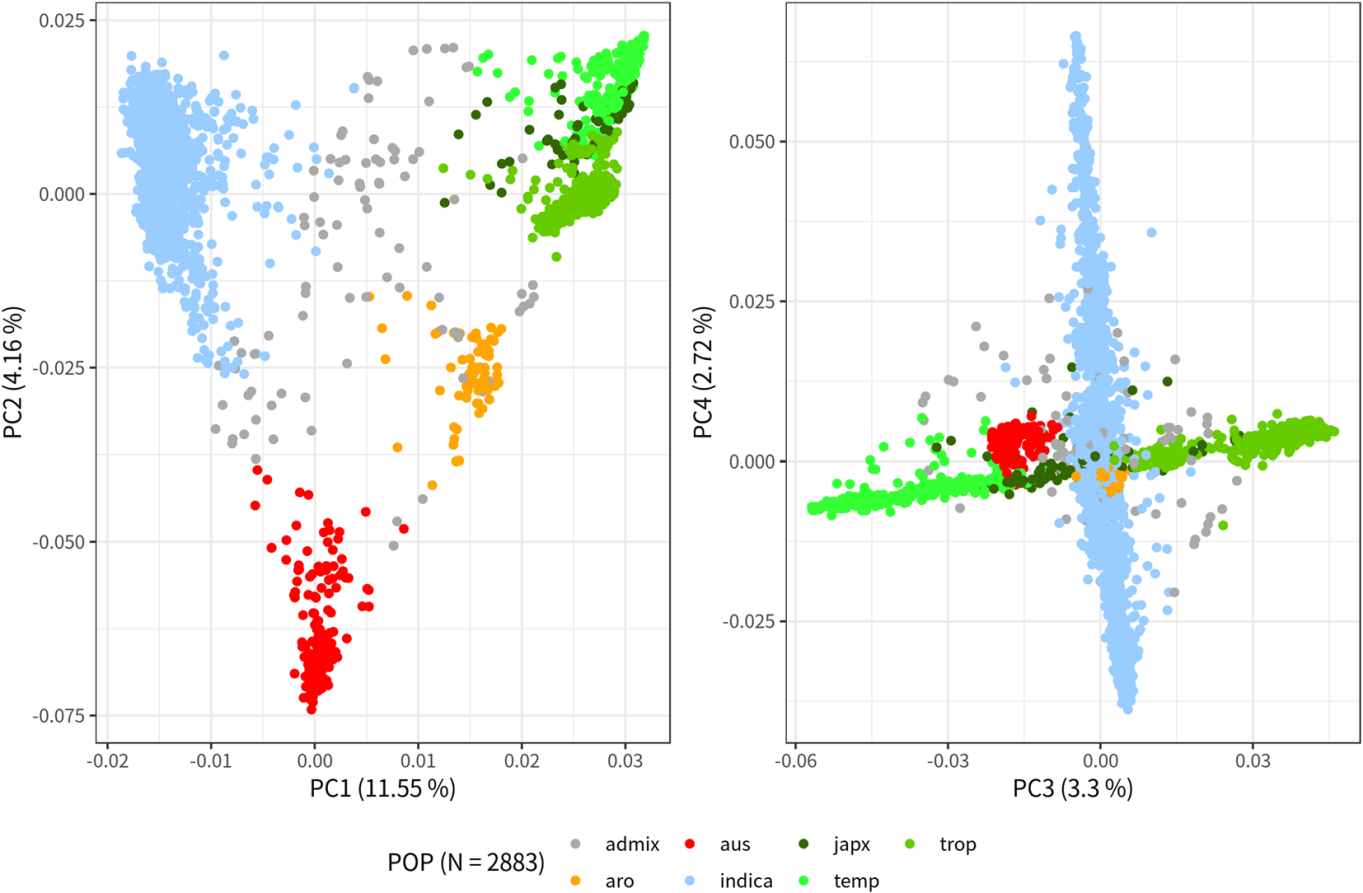
PCA based on 2883 accessions × 14,462 SNPs. Sub-population designation of 2883 accessions was based on Wang et al. (2018): Admix: admixed; aro: *aromatic*; aus: *aus*; indica: *indica*; japx: admixed *japonica*; temp: *temperate japonica*; trop: *tropical japonica*

**Fig. 3:**
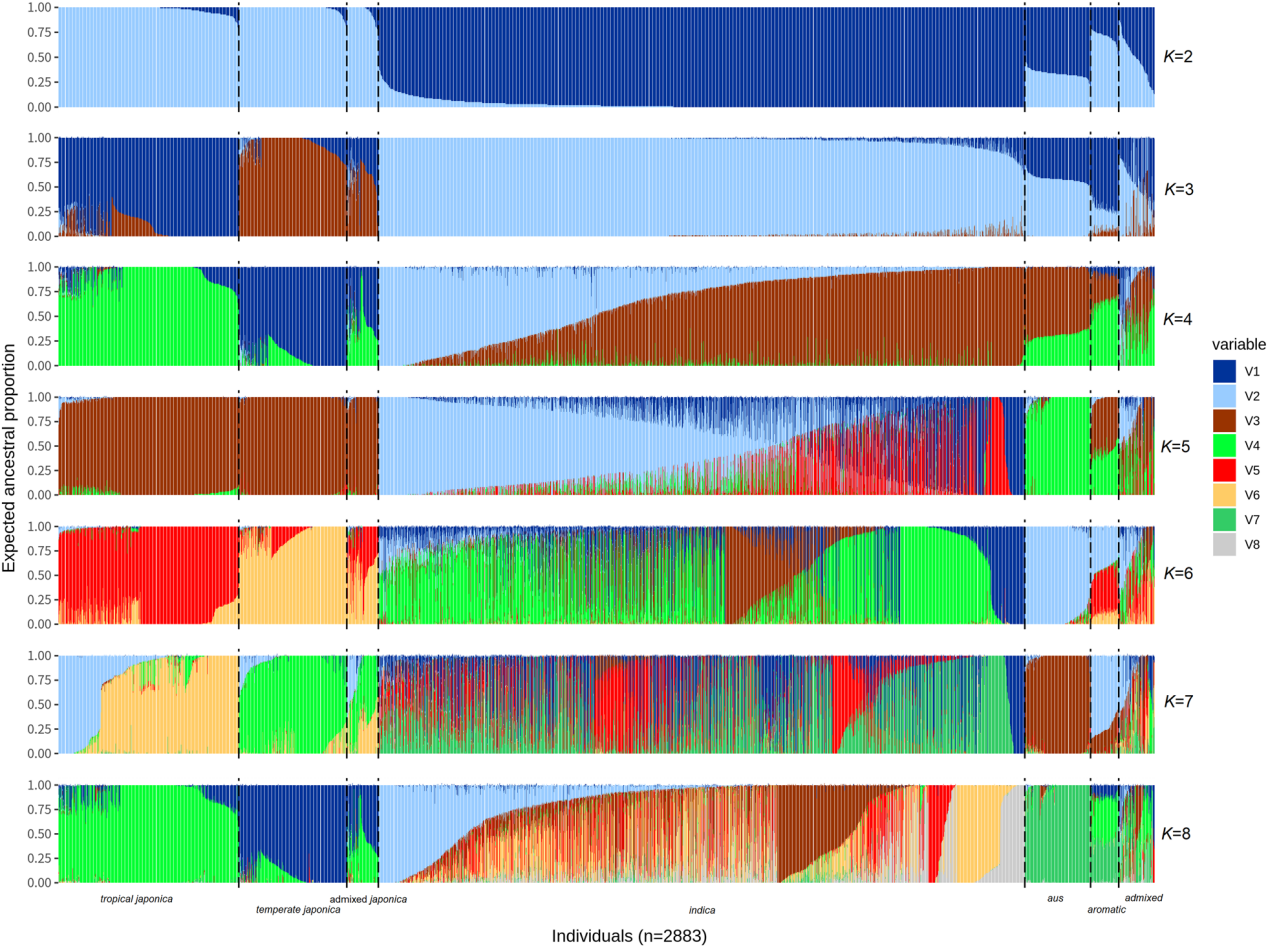
Model-based population structure at K = 2 to 8. Sub-population designation is based on Wang et al. (2018) and are separated by dashed lines

PCA was first conducted to assess population structure of the 2883 accessions after removing some individuals (cf. Methods/Population structure and linkage disequilibrium). The amount of genetic variation explained by PC1 to PC4 was 11.5%, 4.76%, 3.3%, and 2.72%, respectively (Fig. 2). Despite the fact that the first PC explained relatively less variance compared to previous studies (Wang et al. 2014; Zhao et al. 2011), the five main sub-populations, *aromatic*, *aus*, *indica*, *temperate japonica* and *tropical japonica* of Asian rice were clearly distinguished within the sample (Fig. 2). Population structure inferred through the model-based approach was tested for number of sub-populations (K) ranging from 1 to 15. The decision of an appropriate K within a dataset usually relies on a sudden drop in the cross-validation error between K values. Meanwhile, the error estimated in five-fold cross validation decreased smoothly with the increase of K and was minimized at the largest value at K = 15 (Additional file 1: Figure S1). At K = 2, *indica* and *japonica* were clearly distinguished, followed by the separation within the *japonica* group at K = 3 (Fig. 3). At K = 5, *aus* could be distinguished from the other sub-populations, although the separation between *japonica* accessions disappeared, which may be due to the bias of algorithm under the circumstances where strong genetic drift specific to a sub-population was present in the sample, or a sub-population accounted for a large proportion (Marees et al. 2018). *aromatic* could be distinguished from K = 7, meanwhile the *temperate japonica* and a set of *indica* would belong to the same sub-population. Considering the population structure revealed by the PCA and the model-based analysis, as well as information from a previous study using the rice 3K panel (Wang et al. 2018), and the need of a certain sample size to achieve reasonable statistical power for GWAS, we have selected K = 5 for subsequent analysis. Potential admixed accessions were removed for a better control over false positives resulting from hindered stratification or genetic heterogeneity (Korte and Farlow 2013; Tian et al. 2008). A final of 1378 accessions were retained for further analyses: 155 accessions were assigned to *aus*, 696 to *indica*, 152 to *temperate japonica*, and 375 to *tropical japonica*. No *aromatic* accessions passed our criteria of selection for non-admixed individuals (Fig. 3) therefore the *aromatic* sub-population was discarded from subsequent analyses.

**Fig. 4:**
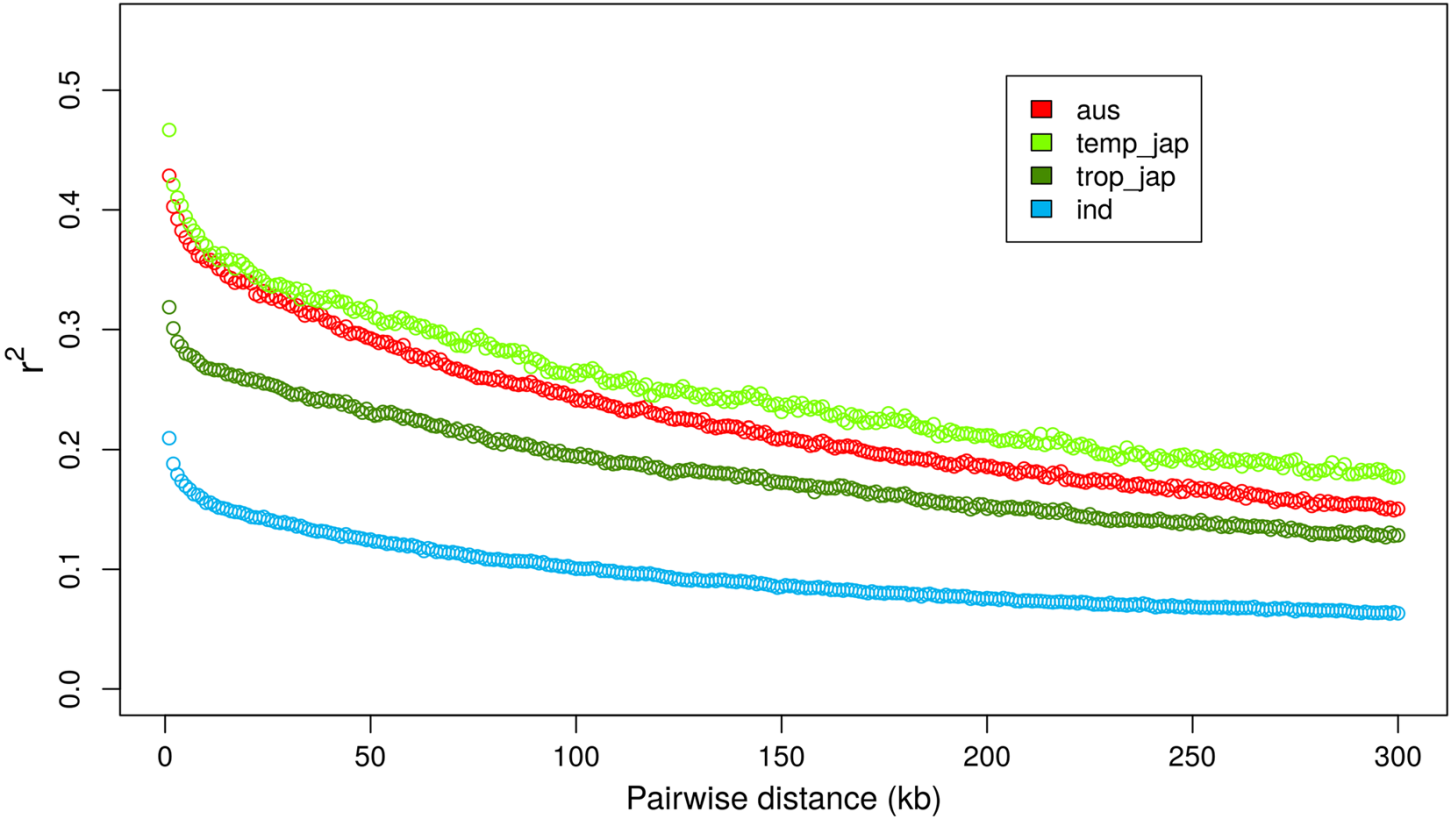
Pair-wise marker linkage disequilibrium (LD) estimation within each sub-population

LD observed in each sub-population was generally lower than that estimated in previous studies (McNally et al. 2009; Xu et al. 2012) (Fig. 4) while the pattern became more congruent with the original study when calculating LD based on another dataset of 4.8 million SNPs that were not LD-pruned (Wang et al. 2018) (Additional file 1: Figure S2). In the core SNP dataset that we used, all sub-populations exhibited an LD decay rate of 100–200 kb at which *r*^*2*^ dropped to half of its maximum value. The sub-population of *temperate japonica* exhibited the shortest LD decay (91 kb), followed by *aus* (146 kb) and *tropical japonica* (157 kb). The mean *r*^*2*^ value observed in *indica* tended to be much lower than other populations, implying almost no LD between markers within the *indica* sub-population. Nevertheless, the genome-wide marker density with such low LD was already high (between 94,005 SNPs for *aus* and 142,836 SNPs for *indica*; cf. Methods/Genome-wide association study) and markers were well distributed along the genome (Additional file 1: Figure S3).

### Trait distribution

**Fig. 5:**
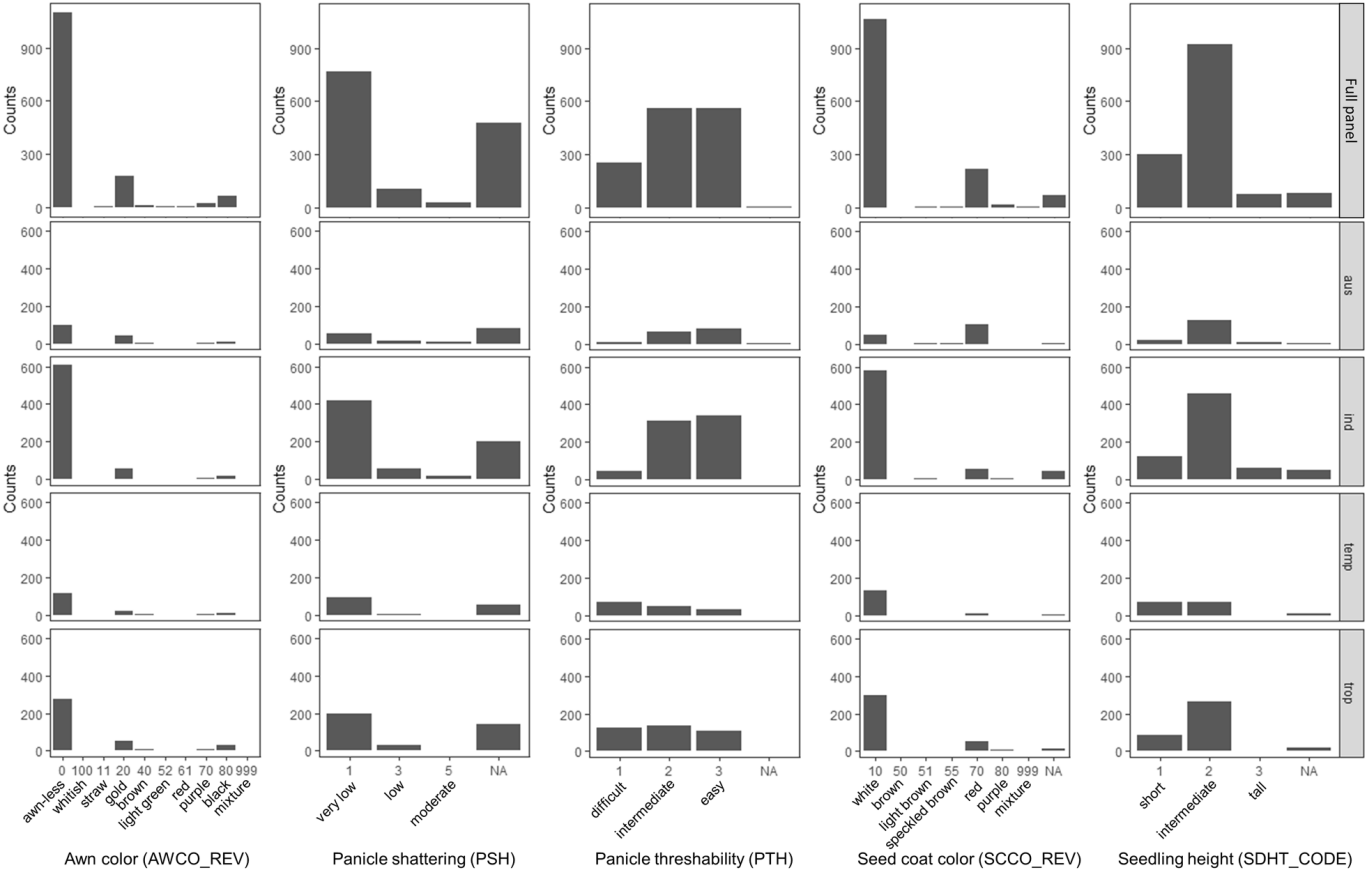
Phenotypic distribution of the five target traits in 1378 rice accessions in the full panel and within each sub-population. For awn color (AWCO_REV), 0, 11, 20, 40, 52, 61, 70, 80, 100, and 999 stand for awn-less, awn with color whitish, straw, gold, brown, light green, red, purple, black, and mixture, respectively. For panicle shattering (PSH), 1 indicates very low shattering (< 1%), 3 indicates low shattering (3%), 5 indicates moderate shattering (15%). For panicle threshability (PTH), 1 indicates difficult for threshing, 2 indicates intermediate for threshing (25% to 50% grains removed), 3 indicates easy for threshing (> 50% grains removed). For seed coat color (SCCO_REV), 10, 50, 51, 55, 70, 80, and 999 stand for white, brown, light brown, speckled brown, red, purple, and mixture, respectively. For seedling height (SDHT_CODE), 1 indicates short seedling (< 30 cm), 2 indicates intermediate seedling (30-59 cm), 3 indicates tall seedling (> 59 cm)

Across the full panel of 1378 accessions, target traits usually had a missing rate smaller than 0.1, except for shattering degree (PSH) which had a missing rate of 0.35 (Fig. 5). Within each sub-population, the distribution of awn color (AWCO_REV), shattering degree (PSH) and seed coat color (SCCO_REV) were highly skewed toward cultivated phenotype since most accessions were improved cultivars, characterized by awn-less panicle, loss of shattering, and white pericarp (Fig. 5). However, there were individuals showing awned panicle and colored pericarp in all sub-populations, especially in *aus* where awn color and seed coat color showed relatively balanced distribution. Panicle threshability (PTH) described here is an agronomic trait distinct from shattering. While shattering is defined as the proportion of grains detached at reproductive stage, threshability is defined as the proportion of grains removed when firmly grasped by hand (IRRI, 2002). Threshability showed relatively balanced distribution within *aus*, *temperate japonica* and *tropical japonica*, whereas most *indica* accessions showed an easy grain removal during threshing process. Many accessions, particularly *indica*, exhibited a low to intermediate (30–59 cm) seedling height at 5-leaf stage.

### GWAS and investigation of trait-associated SNPs

**Fig. 6:**
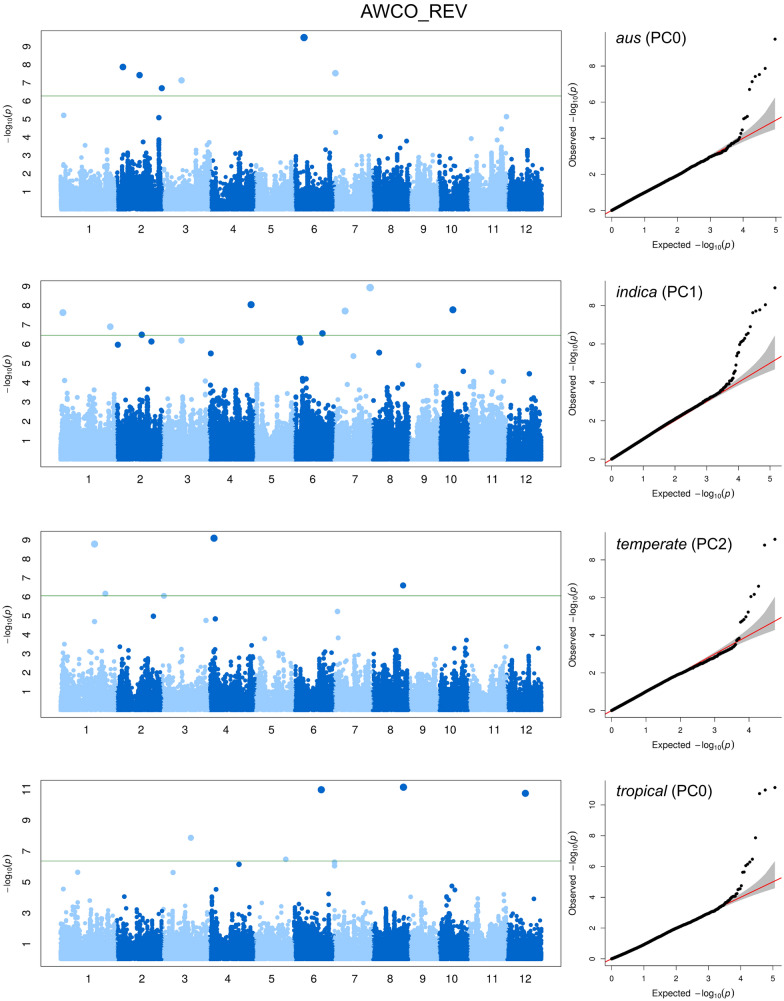
GWAS results for awn color (AWCO_REV) in each sub-population. Manhattan plot was at the left side, quantile–quantile plot was at the right side. From top to bottom: GWAS results for *aus*, *indica*, *temperate japonica*, and *tropical japonica*, the number of PC included in the final model was indicated between parentheses

GWAS were conducted separately in each sub-population given that the existence of population structure may result in spurious associations and that sub-population specific variants may be ignored in a combined population (Wang et al. 2018). There is no single model appropriate for a given trait neither for a given population since the genetic architecture underlying a trait varies between populations. Therefore, model selection is needed for each population × trait combination. Different numbers of PC were included as fixed effects in the final GWAS model for each trait within each sub-population (Fig. 6, Additional file 1: Table S1, Figure S4). As we performed GWAS within sub-populations, most sub-population × trait combinations did not require the inclusion of PC (12 out of 20 sub-population × trait combinations included zero PC). The 8 sub-population × trait combinations which required the inclusion of PC in GWAS model were PSH in *aus* (1 PC), AWCO_REV, PTH, and SDHT_CODE in *indica* (1 PC each), AWCO_REV in *temperate japonica* (2 PC) and PSH, PTH, and SDHT_CODE in *tropical japonica* (1, 2, and 2 PC, respectively).

Overall, 66 trait-associated SNPs were identified over five target traits (Table 1): 23 for awn color (AWCO_REV), 8 for panicle shattering degree (PSH), 3 for panicle threshability (PTH), 19 for seed coat color (SCCO_REV), and 13 for seedling height (SDHT_CODE). All trait-associated SNPs were population-specific. Meanwhile, one region within 26 kb on Chromosome 4 harbored three awn-associated SNPs identified in *aus* and one other awn-associated SNP identified in *temperate japonica*. Three SNPs within 5.3 kb which were close to or inside *Rc* gene on Chromosome 7 were identified from *aus*, *indica*, and *tropical japonica* (Table 1). Twelve out of 66 SNPs, associated with seed coat color, awn color, or panicle shattering degree, fell inside of 11 annotated genes (Table 1). Based on our LD estimation, we further assessed the ± 100 kb region of every trait-associated SNPs in order to identify other potential candidate genes. The number of annotated genes within the ± 100 kb region varied from 8 to 42, most of them were hypothetical protein, of unknown function, or of functions without clear association with the trait of interest. Nevertheless, we have identified four candidate genes close to the trait-associated SNP, including *Rc* (143 bp downstream of S7_6062746 identified within *tropical japonica*), *RAE2* (23.4 kb upstream of S8_24022229 associated to awn), *WOX10* (2.4 kb upstream of S8_25508381 associated to seedling height), and *GRF8* (7.3 kb downstream of S11_20514671 associate to seedling height).

**Table 1:** Trait-associated SNP identified within each sub-population of 3K-RGP

Trait	Pop	SNP	P-value	No. gene ± 100 kb	Candidate genes	Annotation
AWCO_REV	*ind*	S1_2222938	2.34E−08	19		
	*temp*	S1_26642467	1.64E−09	18		
	*temp*	S1_34999638	6.77E−07	25	*Os01g0820700*	*OsWRKY116*
	*ind*	S1_38893251	1.26E−07	32	*Os01g0894500*	Sep15/SelM redox domain containing protein
	*aus*	S2_5126277	1.35E−08	36		
	*aus*	S2_18016425	3.79E−08	15		
	*ind*	S2_20021623	3.27E−07	19		
	*aus*	S2_35221348	1.99E−07	42		
	*aus*	S3_14604626	7.34E−08	26		
	*trop*	S3_22066093	1.37E−08	22		
	*temp*	S4_3569126	8.05E−10	13		
	*ind*	S4_32368836	9.04E−09	40	*Os04g0636500*	BTB domain containing protein
	*trop*	S5_23650885	3.36E−07	30		
	*aus*	S6_7624195	3.23E−10	25		
	*trop*	S6_21261276	1.08E − 11	19		
	*ind*	S6_22232682	2.78E−07	25		
	*aus*	S7_671056	2.97E−08	27		
	*ind*	S7_8538306	1.93E−08	8	*Os07g0253100*	Conserved hypothetical protein
	*ind*	S7_27892037	1.19E−09	36		
	*temp*	S8_23747966	2.51E−07	22		
	*trop*	S8_24022229	7.43E−12	37	*Os08g0485800*	Barwin-related endoglucanase domain containing protein
					*Os08g0485500* (− 23.4 kb)	*RAE2*; Regulation of awn development and elongation
	*ind*	S10_10954998	1.67E−08	16		
	*trop*	S12_14872482	1.84E−11	11		
PSH	*temp*	S2_18492267	1.99E−07	19		
	*temp*	S3_3398933	1.35E−08	34		
	***aus***	**S4_4462336**	**7.23E**−**08**	**21**		
	***aus***	**S4_4462447**	**2.33E**−**07**	**21**		
	***temp***	**S4_4464494**	**1.28E**−**07**	**21**		
	***aus***	**S4_4488660**	**9.51E**−**08**	**21**	***Os04g0166000***	**Conserved hypothetical protein**
	*temp*	S4_5387818	4.08E−07	10		
	*temp*	S12_1917244	5.88E−08	35	*Os12g0139400*	*OsRR10*
PTH	*trop*	S2_24505237	1.22E−07	23		
	*trop*	S7_13096799	5.14E−12	12		
	*trop*	S8_20373414	1.06E−10	20		
SCCO_REV	*ind*	S1_8264505	2.21E−07	30		
	*ind*	S1_14709154	2.38E−14	18		
	*ind*	S1_19343008	2.26E−07	18		
	*trop*	S2_20541410	5.66E−08	10		
	*ind*	S2_35121783	1.35E−07	34	*Os02g0818700*	Similar to tumor-related protein
	*temp*	S3_4766628	8.81E−07	36		
	*temp*	S3_12419022	3.69E−09	32		
	*trop*	S3_16978020	4.30E−07	18		
	*aus*	S3_29290186	4.03E−08	20		
	***trop***	**S7_6062746**	**3.98E**−**54**	**15**	***Os07g0211500*** **(-0.1** **kb)**	***Rc***
	***ind***	**S7_6067855**	**1.15E**−**114**	**15**	***Os07g0211500***	***Rc***
	***aus***	**S7_6068017**	**1.81E**−**40**	**15**	***Os07g0211500***	***Rc***
	*temp*	S7_23073822	7.43E−15	29	*Os07g0571500*	Similar to transmembrane protein 49
	*ind*	S8_8581885	1.39E−08	15		
	*aus*	S8_9105408	4.44E−11	22	*Os08g0249100*	*OsRLCK249*
	*ind*	S9_1298020	2.85E−08	14		
	*temp*	S10_10394027	1.15E−08	13	*Os10g0346300*	*OsLAC17*
	*trop*	S11_17682735	8.45E−09	10		
	*trop*	S11_19853800	6.99E−08	12		
SDHT_CODE	*aus*	S1_15921134	1.38E−07	11		
	*aus*	S1_30508051	1.69E−08	37		
	*temp*	S1_31283533	4.33E−07	27		
	*ind*	S2_19154582	8.69E−08	19		
	*aus*	S2_19678534	3.74E−08	24		
	*aus*	S4_21716120	1.96E−07	35		
	*ind*	S8_8643208	4.52E−13	11		
	*ind*	S8_25508381	2.02E−09	25	*Os08g0242400* (-2.4 kb)	*OsWOX10*
	*ind*	S11_20514671	4.20E−11	20	*Os11g0551900* (+7.3 kb)	*OsGRF8*
	*ind*	S11_24872440	2.62E−11	12		
	*aus*	S12_154948	4.55E−08	26		
	*ind*	S12_365399	6.12E−08	34		
	*ind*	S12_25154542	2.89E−11	26		

The meaning for each trait abbreviation is as follows: AWCO_REV: awn color; PSH: panicle shattering degree; PTH: panicle threshability, SCCO_REV: seed coat color; SDHT_CODE: seedling height. Pop: sub-populations, including *aus*, *indica* (*ind*), *temperate japonica* (*temp*), and *tropical japonica* (*trop*). Markers were named based on their genomic position on the reference sequence (cv Nipponbarre, IRGSP v1) for S[chromosome]_[bp position on the chromosome]. When the trait-associated SNP fell into an annotated gene, the gene name was indicated in the column of “Candidate gene”. If a gene, whose function was related to the trait of interest, fell within ± 100 kb of the trait-associated SNP, the distance between the SNP and the start of the gene is indicated in the parentheses: minus sign means before the SNP, plus sign means after the SNP. Annotation was from RAP-DB. Closely positioned SNPs for which the same genes were identified within the ± 100 kb region were underlined in bold

As our objective was not only to identify trait-associated loci, but also to assess the probability a weedy trait would appear when an individual carries a certain genotype at loci of interests, we inspected the segregation ration between target traits and associated markers for each sub-population × trait combination. We have identified nine SNPs whose allele could indicate with at least 60% of accuracy the target phenotype (Table 2). Here, accuracy is defined as the probability that a phenotype occurs at a given allele. For example, among 145 individuals of *aus*, the frequency of genotype AA at S6_7624195 was 0.74. Among all the individuals carrying AA genotype of S6_7624195, 73.8% were awn-less individuals. The homozygous G at S7_13096799 in *tropical japonica* indicated the tendency of difficult threshing, while the homozygous A, although of lower frequency in the population (0.23), indicated the tendency of easy threshing. Seed coat color could be predicted with an average of 88.7% accuracy by the genotype at target loci: two SNPs identified for *aus*, one SNP for *indica*, and one SNP for *tropical japonica*, located in or in the vicinity of *Rc*, a bHLH protein known to involved in the pigment synthesis at seed coat (Singh et al. 2017; Sweeney et al. 2006) for which we will provide a detail description further. SNPs whose genotypes could predict with a certain accuracy seedling height were identified for *indica* (S11_24872440) and *temperate japonica* (S1_31283533).

**Table 2:** Phenotype prediction accuracy based on genotype of selected trait-associated SNPs

Trait	Pop	No. Ind	SNP	Genotype	Freq	Prediction accuracy
AWN	*aus*	145	S6_7624195	AA	0.74	73.8% awn-less
				GG	0.26	68.4% awned
AWN	*trop*	375	S8_24022229	GG	0.9	80.1% awn-less
				AA	0.1	89.1% awned
PTH	*trop*	378	S7_13096799	GG	0.76	73.4% difficult (41.6%) to intermediate (31.8) threshing
				AA	0.23	90.9% easy (38.6%) to intermediate (52.2%) threshing
SCCO	*aus*	153	S7_6068017	CC	0.68	96.1% colored seed coat
				AA	0.31	93.8% white seed coat
SCCO	*aus*	153	S8_9105408	GG	0.65	86% colored seed coat
				AA	0.33	66.7% white seed coat
SCCO	*ind*	645	S7_6067855	GG	0.92	96.1% white seed coat
				AA	0.07	91.5% colored seed coat
SCCO	*trop*	362	S7_6062746	TT	0.9	90.5% white seed coat
				GG	0.1	88.9% colored seed coat
SDHT	*ind*	641	S11_24872440	CC	0.85	92.1% short (21%) to intermediate (70%) seedling
				TT	0.13	95.2% intermediate (71%) to tall (23%) seedling
SDHT	*temp*	137	S1_31283533	AA	0.75	62.1% short seedling
				GG	0.25	94% intermediate seedling

The meaning for each trait abbreviation is as follows: AWN: awn color; PTH: panicle threshability, SCCO: seed coat color; SDHT: seedling height. Sub-population × trait combinations are shown in the table if the genotype of trait-associated SNP can predict the phenotype with at least 60% of chance to accuracy. Otherwise we do not present the Sub-population × trait combinations for a clear and concise presentation

**Fig. 7:**
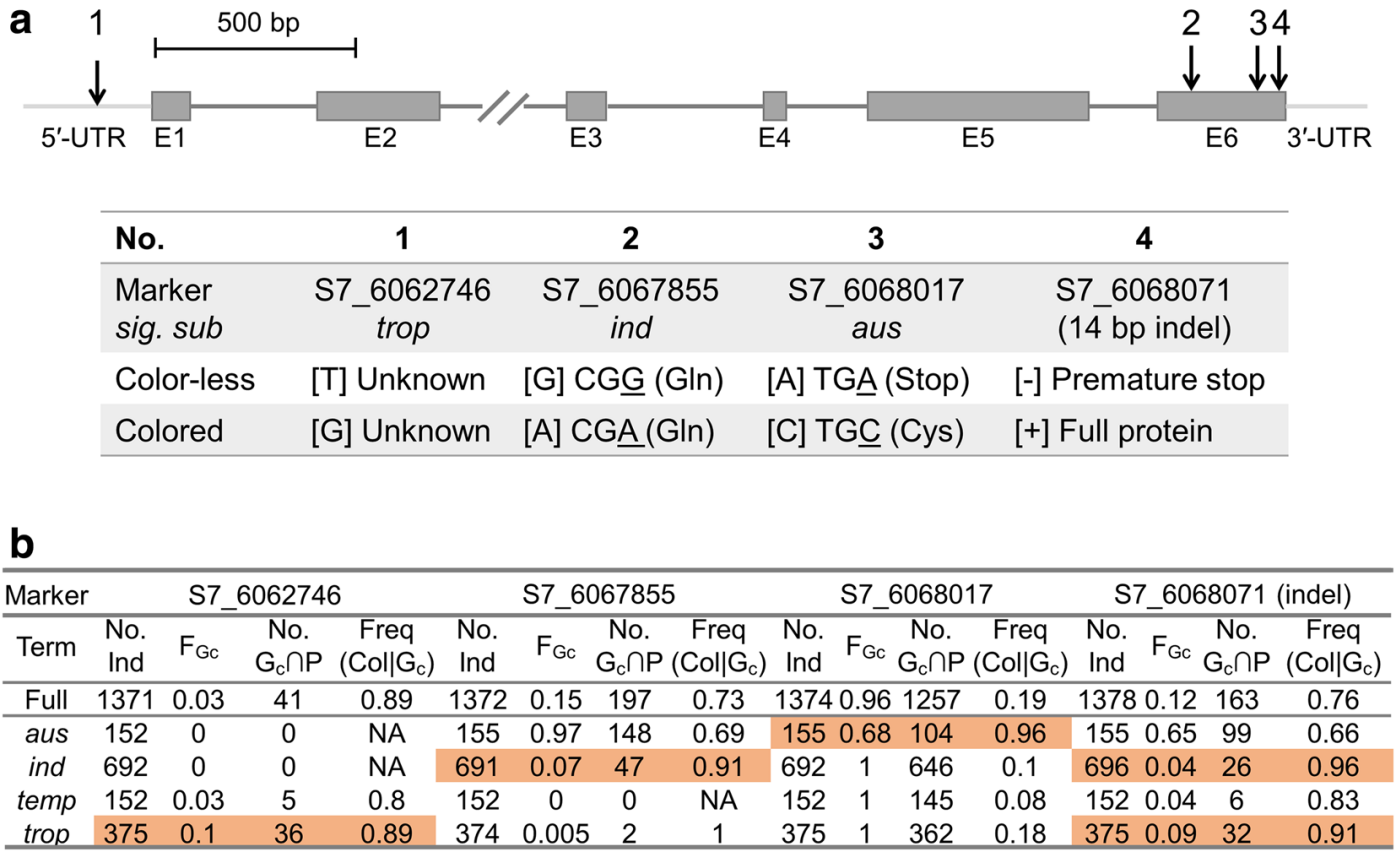
Relationship between genotype and phenotype for seed coat/pericarp color-associated SNP inside or close to the known candidate gene *Rc*. **a** Position of the SNPs were indicated by arrows on the diagram of the structure of *Rc*. E1 to E6 stand for exon 1 to exon 6. The gene structure of *Rc* is proportional to the actual base-pair length, except for intron 2 which is ca. 2.7 kb. *sig. sub* indicates the sub-population from which the corresponding seed coat color-associated SNP was identified. The color-less or colored seed coat-associated allele, as well as the associated functional alteration were indicated. **b** For each trait-associated marker, its number of individuals used for the calculation of frequency of individuals carrying the “colored” genotype (F_Gc_), the number of individuals carrying the “colored” genotype which had seed coat color information (No. G_c_∩P), and the frequency that the individuals carrying colored genotype had indeed colored seed coat (Freq(Col | G_c_)). Within a sub-population, SNPs whose alleles correspond well to phenotypes are highlighted in orange. Meanwhile, only SNP × sub-population combinations with No. Gc∩P exceeding 20 are highlighted

The high prediction accuracy of SNP genotypes for seed coat color drove us to investigate in detail these seed coat color-associated SNPs located within or in the vicinity of *Rc* on chromosome 7 (Fig. 7) and we have included the functional 14-bp InDel for a comparison. S7_6062746 was identified in the sub-population of *tropical japonica* and was located in the 5′- untranslated region (5′-UTR). The two alleles T and G of this SNP had no functional evidence yet. S7_6067855 was identified within *indica* and the two alleles were synonymous mutation (Fig. 7a). S7_6068017 was identified within *aus* whose color-less allele, A, would cause an early stop codon and lead to an incomplete protein product. This allele has been previously assigned as *rc*-*s* allele (Singh et al. 2017; Sweeney et al. 2006). Further, we verified the frequency of accessions carrying the “colored” allele and the frequency of individuals carrying “colored” allele showing indeed “colored phenotype” (Freq (Col|G_c_) in Fig. 7b) in the full panel (1378 accessions) and within each sub-panel (parallel information for “color-less” allele is provided in Table S2). We observed that, although the genotype of the 14-bp InDel generally explained well the occurrence of red seed coat, the “colored” allele of SNPs identified for each sub-population explained even better the occurrence of red seed coat for sub-populations. The most striking case was related to *aus*: the frequency of individuals carrying the 14-bp insertion showing effective red seed coat in *aus* was 0.66, while 96% of individuals carrying the “colored” allele of S7_6068017 possessed colored seed coat in *aus*. No seed coat color-associated SNP was identified for the sub-population of *temperate japonica* which should be a result of too few individuals exhibiting colored seed coat.

### Comparison between 3K-RGP and weedy rice

**Fig. 8:**
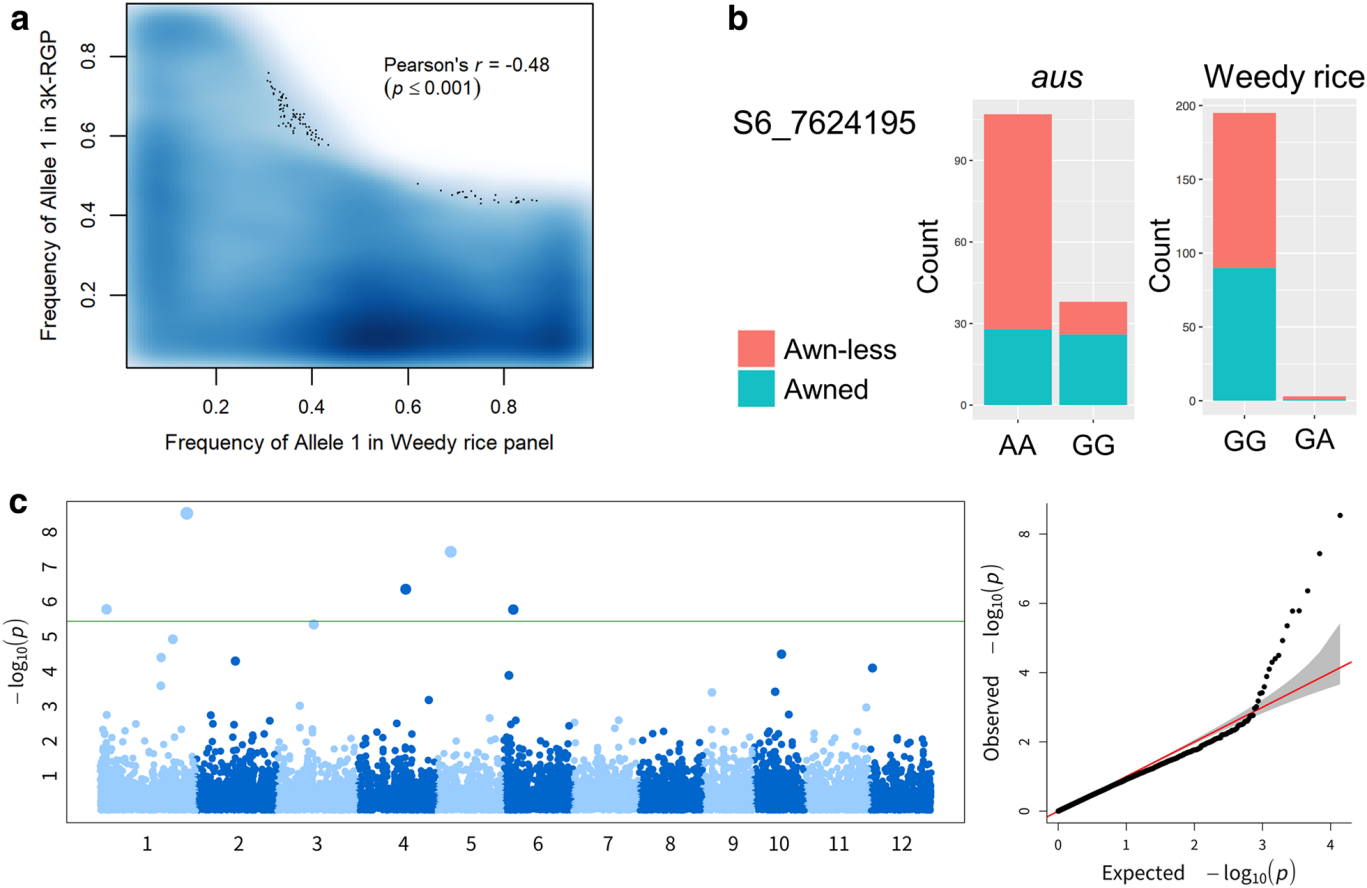
Exploration on weedy rice data. **a** Distribution of allele frequency of a given allele between 41,044 common SNPs (MAF ≥ 0.05) between weedy rice panel and 3K-RGP. High density is indicated by darker color; black dots are individual comparison. **b** Phenotype *vs*. genotype segregation of S6_7624195 in *aus* and in weedy rice panel. **c** GWAS results of weedy rice panel. The first two PC based on genome-wide SNPs were included in the GWAS model to control for population structure. The green horizontal line showed the Bonferroni threshold at α = 0.05

In addition to the analysis using the 3K-RGP data, we attempted to compare our results with publicly available WR data. For this purpose, we have obtained sequence reads of 205 WR accessions from two studies (Li et al. 2017; Qiu et al. 2017) which provided both phenotypic and genotypic data. We identified SNPs of WR panel by aligning WR sequence reads to the reference genome (cv. Nipponbare, IRGSP-1.0). After removing monomorphic and low MAF SNPs (MAF < 0.05), we obtained 71,343 polymorphic SNPs for WR panel, among which 41,044 were also found in the 3K-RGP dataset. Detailed observation on the 41,044 common markers revealed different allele profile between the two panels: the minor allele between panels were different, and the allele frequency of the same allele between the two panels was not positively correlated (Pearson’s *r* = − 0.48, p-value < 0.001; Fig. 8a). In terms of phenotypic data, only the presence and absence of awn was available for the WR panel. Therefore, we examined first whether the phenotype–genotype segregation of the two awn-associated explanatory SNPs identified in 3K-RGP (Table 2) could explain the phenotype–genotype relationship in the WR panel. S8_24022229 was absent from the WR panel, while for S6_7624195, no individual carried the “awn-less” genotype (AA); only three individuals carried allele A in heterozygous state (Fig. 8b). We have also conducted GWAS on WR panel (Fig. 8c, Table 3) whose profile and significant SNPs were different from that of 3K-RGP (Fig. 6, Table 1).

**Table 3:** Awn-associated SNPs identified within WR panel

SNP	P.value	No. Gene ± 100 kb	Candidate genes	Annotation
S1_2958149	1.65E−06	42	*Os01g0155000*	Alpha/beta hydrolase fold-3 domain containing protein
S1_38916854	2.89E−09	34	*Os01g0895200*	DOMON related domain containing protein
S4_21610862	4.36E−07	27	*Os04g0434600*	Similar to OSIGBa0102D10.3 protein
S5_6339023	3.68E−08	20		
S6_4425573	1.67E−06	31	*Os06g0187700*	Conserved hypothetical protein

Markers were named based on their genomic position on the reference sequence (cv Nipponbarre, IRGSP v1) for S[chromosome]_[bp position on the chromosome]. When the trait-associated SNP fell into an annotated gene, the gene name was indicated in the column of “Candidate gene”. Annotation was from RAP-DB

## Discussion

The aim of this study was to make use of publicly available data to explore the genetics of “weedy” traits, or, in other words, traits related to domestication. The main dataset that we used were subsets from the 404K core SNPs of the 3K-RGP. We have chosen the 404 core SNP dataset considering both marker density and computational efficiency. Population structure analyses clearly showed the presence of sub-populations within the germplasm in spite of the fact that the model-based approach did not provide a clear-cut K value for the number of sub-populations and prior knowledge was needed for such determination. Knowledge on population structure on a collection of germplasm is particularly important for the subsequent genetic analyses, especially GWAS. It has been demonstrated in plants that population structure could interfere in the identification of trait-associated markers (Alonso-Blanco et al. 2016; Morris et al. 2013; Zhao et al. 2011). Therefore, we have used a relatively stringent criterion (sub-population genome belonging ≥ 0.8) to define sub-populations. As such, no individuals from *aromatic* sub-population were retained for our analysis since they appeared as the results of hybridization between *aus* and *japonica* (Civáň et al. 2015). Our approach seemed to be effective since the inclusion of PC to correct for population structure in GWAS model was mostly unnecessary (Additional file 1: Table S1) and the inclusion of PC for some sub-population × trait combinations could be explained by the fact that weak structure did existed within the sub-populations that we defined (Wang et al. 2018).

Self-pollinated feature of cultivated rice often favors the maintenance of long-range LD (McNally et al. 2009; Zhao et al. 2011). Genome-wide LD decay in cultivated rice has been estimated at a 100–300 kb range across different sub-populations (McNally et al. 2009; Zhao et al. 2011). The relatively low LD value observed in our study should be due to the fact that the 404K core SNP was previously LD-pruned, yet the trend of the LD decay remained similar when we estimated the LD using the 4.8 million un-pruned dataset (Additional file 1: Figure S2).

We used GWAS to detect trait-associated SNPs within each sub-population. Relatively few trait-associated SNPs were identified compared to the number of SNPs used for GWAS. One possible reason was that the available phenotypic data were categorical data which reduced the variation and information of quantitative traits. The other possible reason was that the phenotypic distributions were skewed towards cultivated types, such as absence of awn, low degree of shattering, color-less seed coat, and short stature (Fig. 5). A more balanced phenotypic distribution would help in the estimation of allelic contrast within a marker. Eleven trait-associated SNPs fell into an annotated gene, while four other putative candidate genes were identified using an average LD decay length (± 100 kb). They were actually closer to the putative candidate (within ± 25 kb) compared to the average LD decay. We have observed the same phenomenon in another study where most promising candidate genes were identified within ± 10 kb of the significant SNP (Ma et al. unpublished data). Among the candidate genes identified in this study, the most promising one is *Rc*, a gene encoding a bHLH protein controlling the proanthocyanidin synthesis in rice, a major player in seed coat color formation (Furukawa et al. 2007; Sweeney et al. 2006). A 14-bp deletion within exon 6 of *Rc*, and a shorter *Rc* transcript was found to correlate to the color-less seed coat (Sweeney et al. 2006), which was further confirmed through functional validation that *Rc* did code for a bHLH protein and its 14-bp InDel contribute to the color-less seed coat phenotype (Furukawa et al. 2007). Among the 1378 3K-RGP individuals selected for our study, 163 carried the 14-bp “red” allele of which 76% of individual showed effective red seed coat (Fig. 7). Our detailed analysis showed that the 14-bp “red” allele correlated relatively well to the occurrence of red seed coat within *indica*, *temperate japonica*, and *tropical japonica*, although the number of individuals carrying the red allele was small for the three populations, therefore less representative to a larger scale (Fig. 7). There are other genes known to be involved in seed coat color formation in rice, such as *Rd*, coding for an enzyme of the proanthocyanidin biosynthetic pathway (Furukawa et al. 2007). This could explain that although polymorphisms in *Rc* correlate well with seed coat color but cannot predict 100% the phenotype. Meanwhile, for each sub-population, the trait-associated SNPs identified in our study were more representative to explain the occurrence of red seed coat both in terms of number of individuals for the analysis (No. G_c_ ∩ P in Fig. 7) and the frequency of “red” allele carrying individuals with effective red phenotype (Freq(Col | G_c_) in Fig. 7). The most striking case was S7_6068017 identified in *aus* whose genotype could explained close to the total occurrence of red seed coat. The A allele of S7_6068017 was previous identified as *rc*-*s* allele (Sweeney et al. 2006) and has been noticed to be present at moderate frequency in *aus* (five out of nine white seed coat individuals) and in *aromatic* (two out of 17 white seed coat individuals) but not at all in *indica*, *temperate* or *tropical japonica* (Sweeney et al. 2007). Using a larger sample, we found that both alleles of S7_6068017 were presented in a relative balanced frequency in *aus*. As red seed coat is widely observed for WR (Huang et al. 2018; Li et al. 2017; Qiu et al. 2017), our results on *Rc* could be used to diagnose in part the origin of WR occurred in the field. Some significant trait-associated SNPs were in the vicinity of other candidate genes: the most significant awn color-associated SNP identified in *tropical japonica*, S8_24022229 (Table 1), was 23.4 kb downstream *RAE2*, which was involved in awn development and grain size in Asian rice (Bessho-Uehara et al. 2016). For seedling height, two associated SNPs were found to be close to two candidate genes: S8_25508381 was 2.4 kb downstream of *WOX10*, while S11_20514671 was upstream of *GRF8*. *WOX10* belongs to *WUSCHEL* (*WUS*)-related Homeobox (*WOX*) gene family which have been shown to coordinate gene expression both in shoot and root meristem in Arabidopsis (Haecker et al. 2004). *OsWOX11* was shown to involve in the activation of rice crown root emergence and growth, related to seedling growth. On the other hand, *GRF8* belongs to growth-regulating factor (GRF) family, a plant specific transcription factor. GRF are involved in various plant developmental processes and take part in the coordination of growth under adverse environmental conditions (Omidbakhshfard et al. 2015). Therefore, it can be a good candidate of seedling vigor, an important characteristic for weeds.

Since our work using the data from 3K-RGP was to pave the way for our understanding of the WR occurrence, we have also obtained WR data from public resources to get a first glance of the usefulness of our results from cultivated pool. Although our most promising diagnostic markers were for seed coat color, the common trait from WR panel was the presence/absence of awn, which restricted our analysis. A substantial number of markers were in common between the 3K-RGP panel and the WR panel, but when we investigated quality-filtered markers, allele frequency behaved differently between the two panels, as well as the genotype vs. phenotype segregation and, the genomic position of awn-associated markers (Fig. 8). Indeed, the genotypic data showed that the WR accessions did not grouped with the cultivated rice from the 3K-RGP (data not shown) which implied a different evolution history of the two panels. Such differences did not discredit the result from each panel. Instead, they remind us that although publicly available data is a great tool to explore plant genetics and genomics, certain limits remain and data from complementary laboratory experiment will always be necessary to validate the dry-lab results.

## Conclusion

We have analyzed publicly available data to explore the genetic architecture of weediness-related traits on cultivated pool from 3K-RGP. After assessing population structure and LD, we have performed GWAS within each sub-population and have identified trait-associated markers. Candidate genes were identified for weediness-related traits and the most significant were SNPs from *Rc* on chromosome 7. We have shown that *rc*-*s* was particularly abundant in *aus* and that *Rc* alleles could therefore serve as diagnostic markers to assess the origin of weedy rice in the field, especially to understand the occurrence of colored seed coat. Further, we have compared the data from 3K-RGP to another publicly available data of WR. The constitution of the two panels was so different that their results did not converge toward the same direction. This work showed the potential of publicly available data but also remind the indispensable validation by lab experiments.

## Supplementary information

**Additional file 1: Table S1.** Number of PC included in the final GWAS model. **Table S2.** Relationship between genotype and phenotype for seed coat/pericarp “color-less” allele of SNPs inside or close to the known candidate gene *Rc*. **Figure S1.** cross-validation error for K = 1 to 15. **Figure S2.** LD decay based on 4.8 M SNPs. **Figure S3.** Genome-wide marker distribution for each sup-population. **Figure S4.** Manhattan plots and Q-Q plots for final GWAS model for different sub-population × trait combination.

## Data Availability

The data used and analyzed for the current study can be obtained from the corresponding author.

## Abbreviations

3K-RGP: 3000 Rice Genome Project
BHA: Black-hull awned
GWAS: Genome-wide association analysis
LD: Linkage disequilibrium
PCA: Principle component analysis
SH: Straw awn-less
SNP: Single nucleotide polymorphism
WR: Weedy rice
