# Supplementary material for: Explore the genetics of weedy traits using rice 3K database
Source: Bot Stud. 2021 Jan 12;62:2. doi: 10.1186/s40529-020-00309-y (PMC7801593; doi:10.1186/s40529-020-00309-y)
Supplement: Supplementary file 1 — Additional file 1: Table S1. Number of PC included in the final GWAS model. Table S2. Relationship between genotype and phenotype for seed coat/pericarp “color-less” allele of SNPs inside or close to the known candidate gene Rc. Figure S1. cross-validation error for K = 1 to 15. Figure S2. LD decay based on 4.8 M SNPs. Figure S3. Genome-wide marker distribution for each sup-population. Figure S4. Manhattan plots and Q-Q plots for final GWAS model for different sub-population × trait combination. [file 40529_2020_309_MOESM1_ESM.pdf]

Table S1. No. of PC included in the final GWAS model for each sub-population × trait combination.

	AWCO_REV	PSH	PTH	SCCO_REV	SDHT_CODE
aus	0	1	0	0	0
indica	1	0	1	0	1
temperate japonica	2	0	0	0	0
tropical japonica	0	1	2	0	2

Table S2. Relationship between genotype and phenotype for seed coat/pericarp "color-less" allele of SNPs inside or close to the known candidate gene *Rc*.

Marker	S7_6062746				S7_6067855				S7_6068017				S7_6068071 (indel)			
Term	No. Ind	FGcL	No. GcL∩P	Freq (CL|GcL)	No. Ind	FGcL	No. GcL∩P	Freq (CL|GcL)	No. Ind	FGcL	No. GcL∩P	Freq (CL|GcL)	No. Ind	FGcL	No. GcL∩P	Freq (CL|GcL)
Full	1371	0.97	1261	0.84	1372	0.85	1104	0.86	1374	0.03	48	0.94	1378	0.87	1143	0.90
aus	152	1	150	0.34	155	0.03	4	0.75	155	0.31	48	0.94	155	0.34	52	0.27
ind	692	1	646	0.90	691	0.92	596	0.96	692	0	0	NA	696	0.96	622	0.93
temp	152	0.97	140	0.95	152	1	145	0.92	152	0	0	NA	152	0.96	139	0.96
trop	375	0.9	325	0.90	374	0.99	359	0.83	375	0	0	NA	375	0.91	330	0.89

For each trait-associated marker, its number of individuals used for the calculation of frequency of individuals carrying the "color-less" genotype (FGcL), the number of individuals carrying the "color-less" genotype which had seed coat color information (No. GcL∩P), and the frequency that the individuals carrying color-less genotype had indeed color-less seed coat (Freq(CL | GcL)).

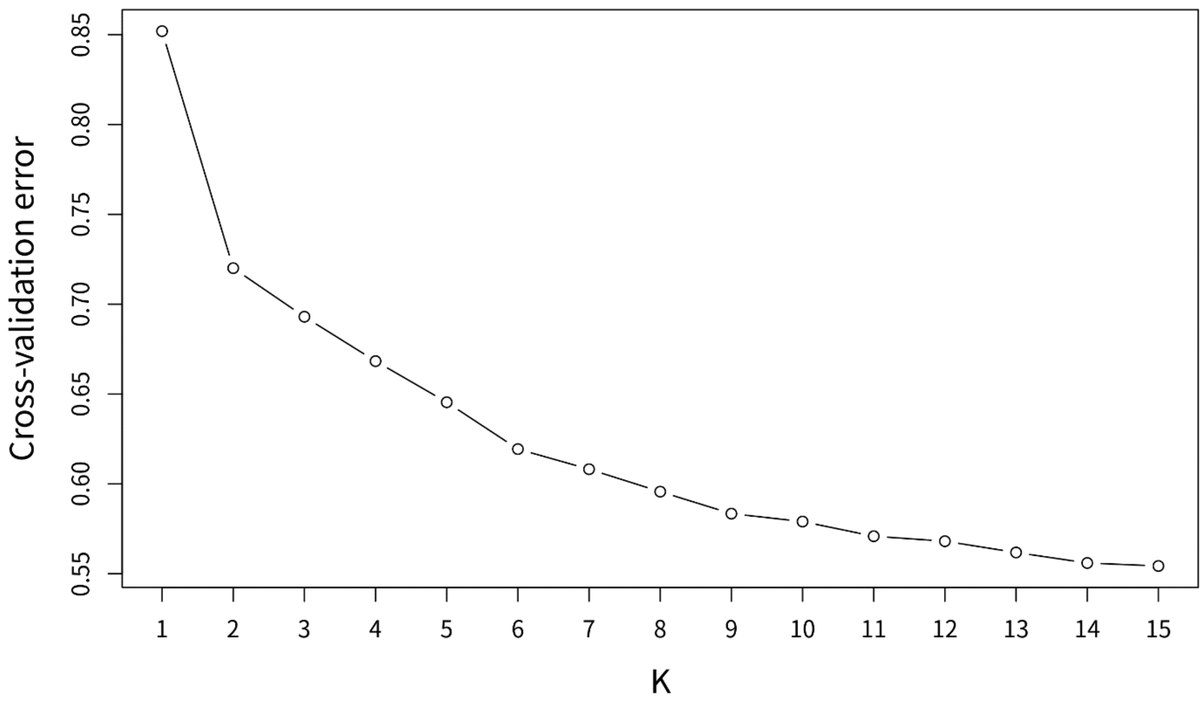

Figure S1. Cross-validation error for K = 1 to 15.

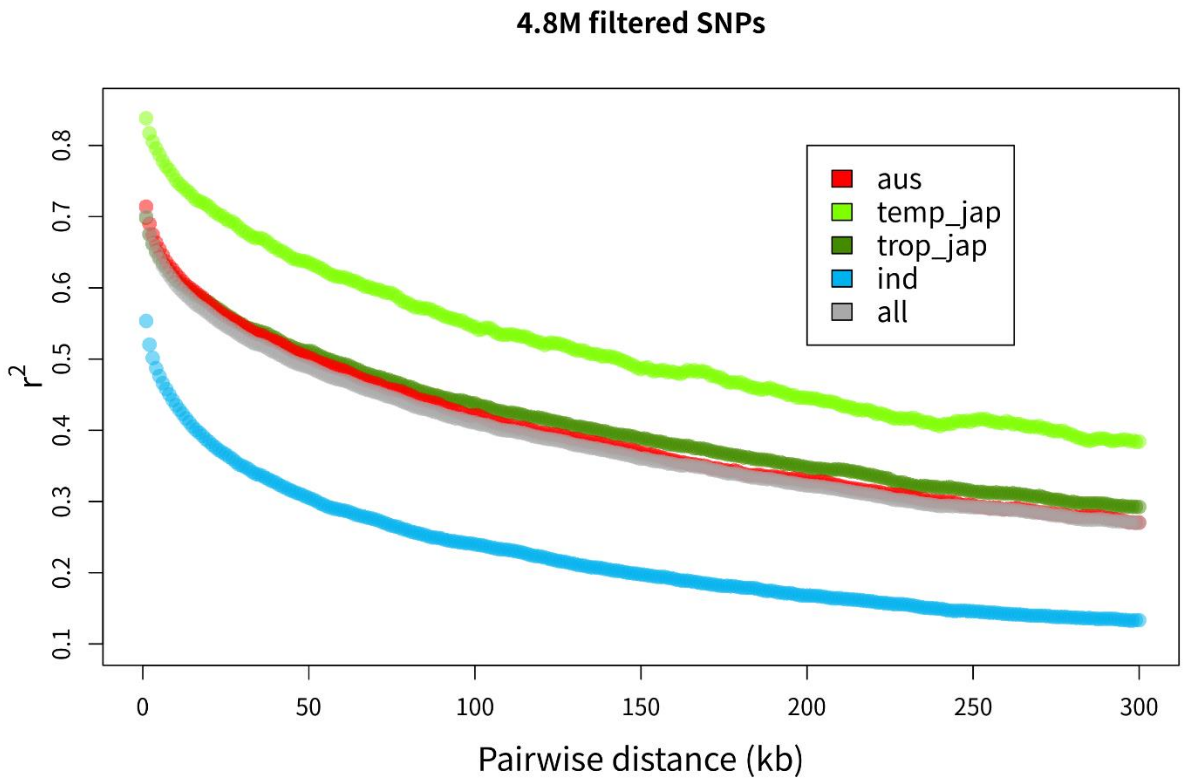

Figure S2. LD decay based on 4.8 M SNPs.

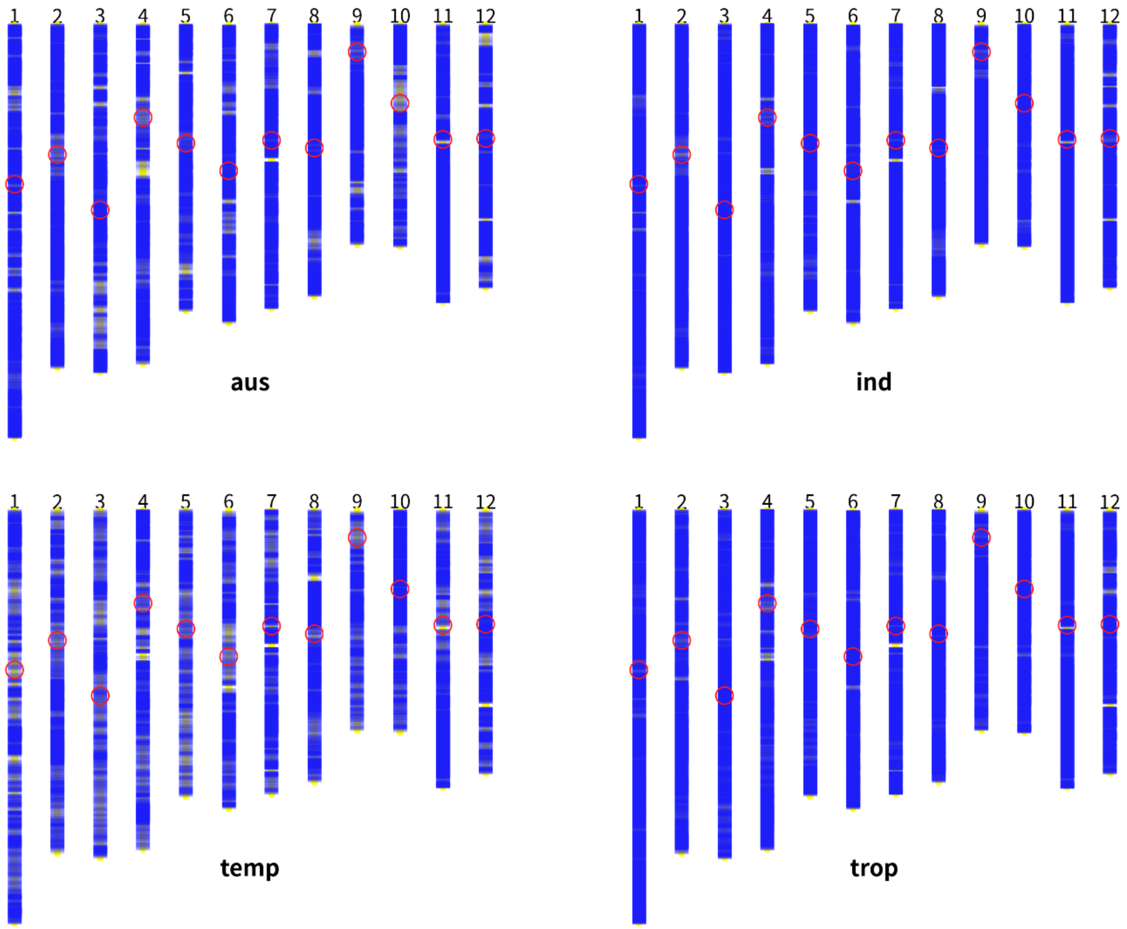

Figure S3. Genome-wide marker distribution for each sup-population.

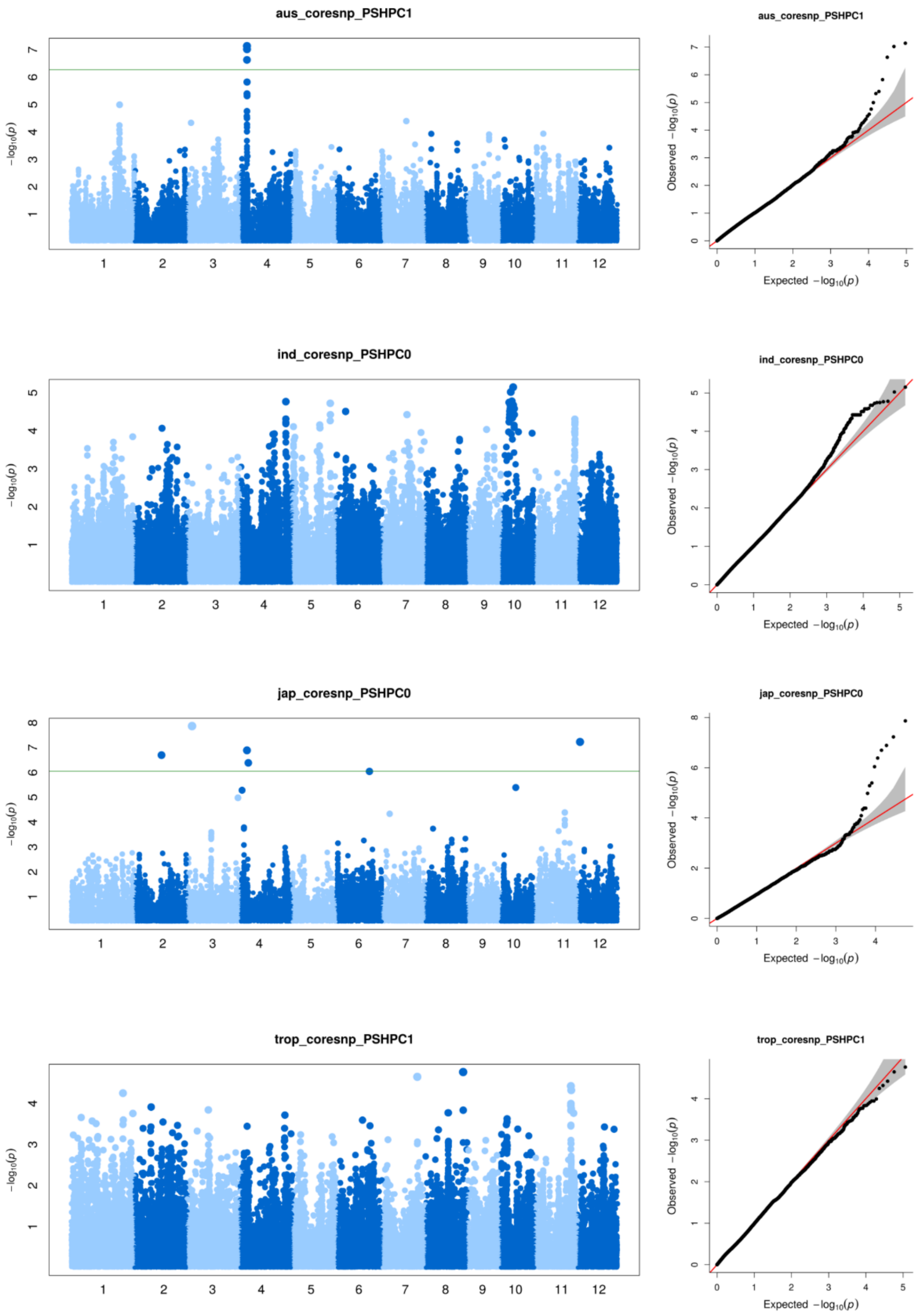

Figure S4 Manhattan plots and Q-Q plots for final GWAS model for different sub-population × trait combinations.

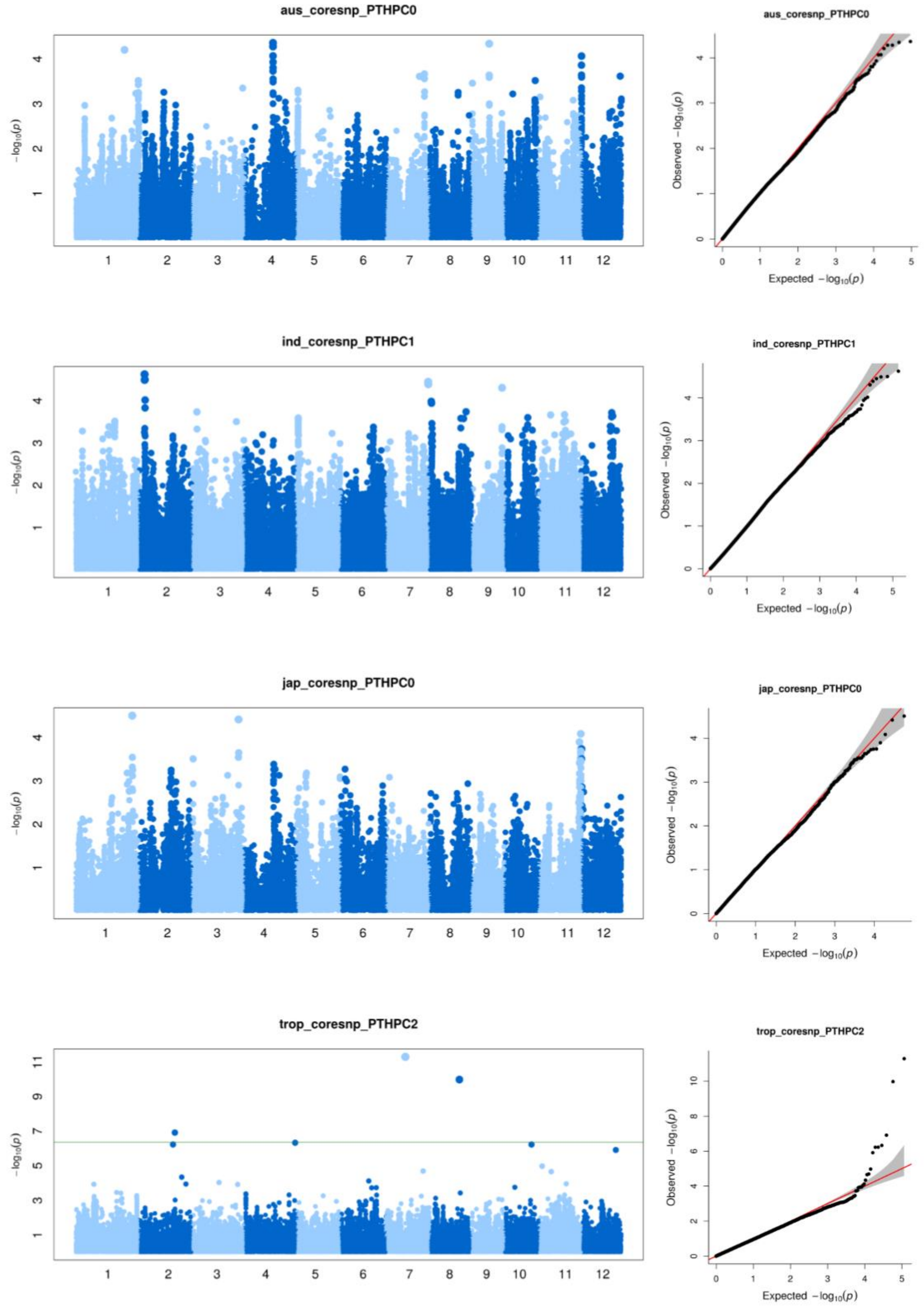

Figure S4 (continued)

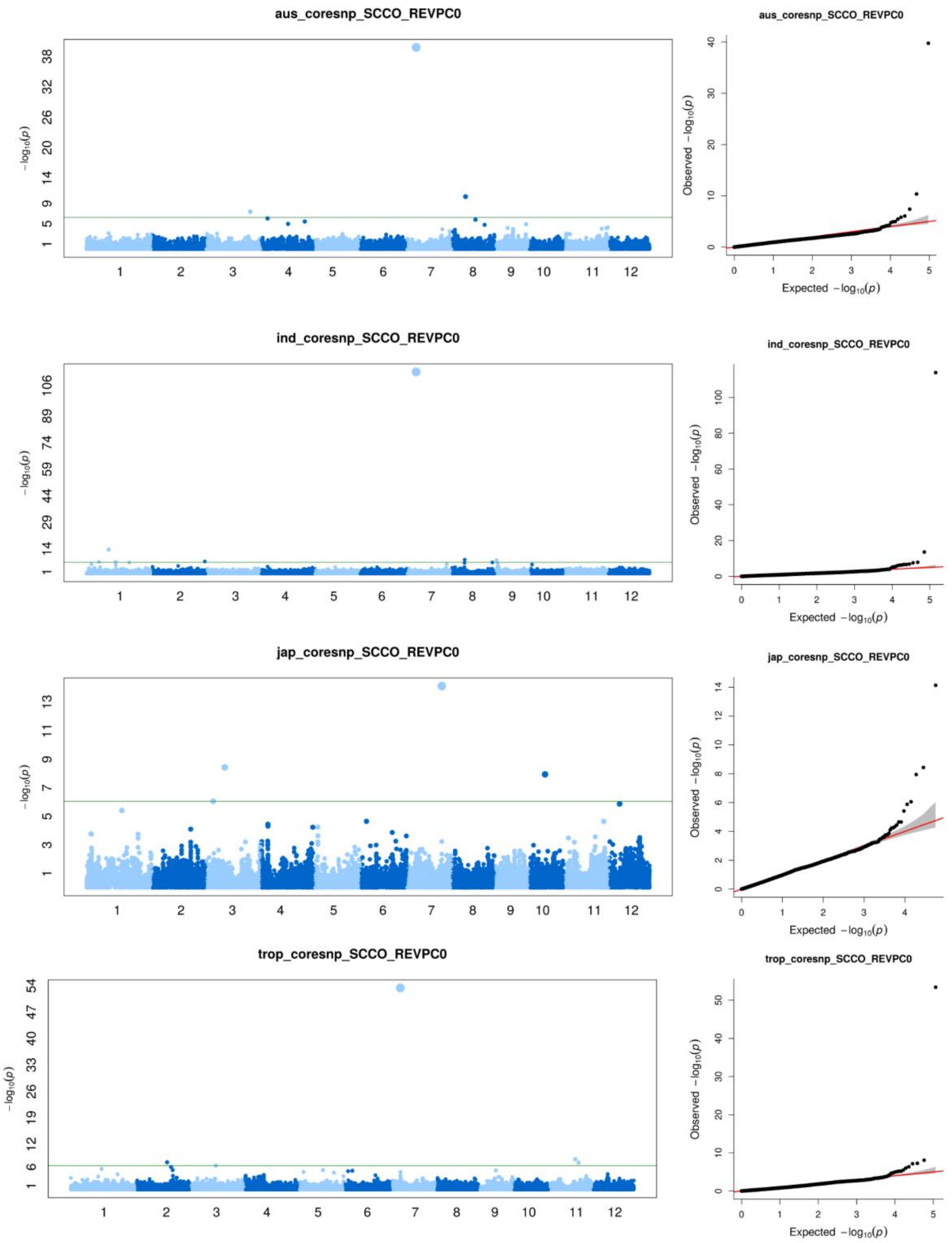

Figure S4 (continued)

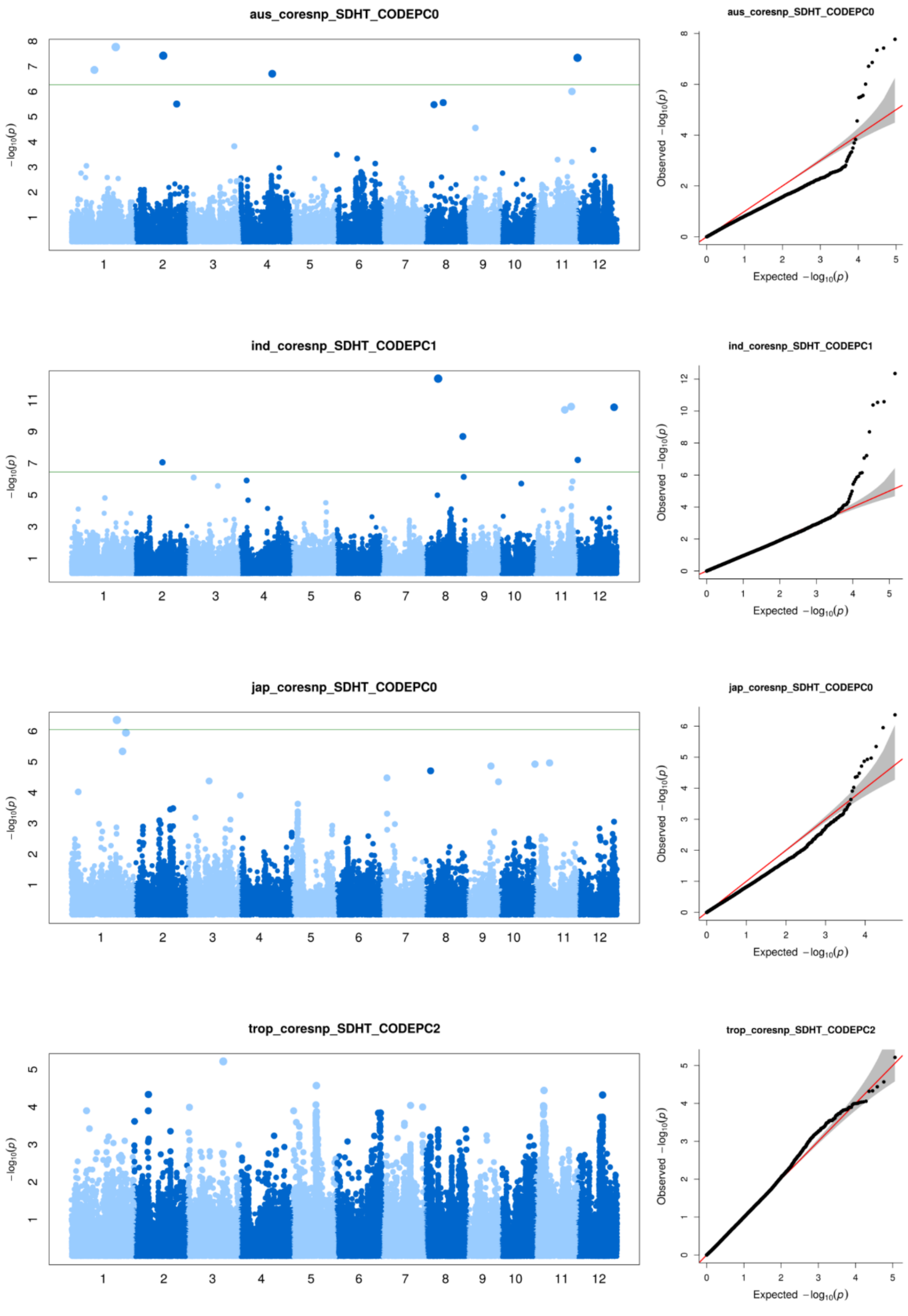

Figure S4 (continued)
